# Moderated-mediation analysis of multimorbidity and health-related quality of life among the Chinese elderly: The role of functional status and cognitive function

**DOI:** 10.3389/fpsyg.2022.978488

**Published:** 2022-11-08

**Authors:** Tongxing Li, Wei Hu, Liang Zhou, Liuming Peng, Lei Cao, Zhaolong Feng, Qida He, Jiadong Chu, Xuanli Chen, Siyuan Liu, Qiang Han, Na Sun, Yueping Shen

**Affiliations:** ^1^Department of Epidemiology and Biostatistics, School of Public Health, Medical College of Soochow University, Suzhou, China; ^2^Department of Chronic Noncommunicable Diseases, Liyang Center for Disease Control and Prevention, Liyang, China

**Keywords:** multimorbidity, health-related quality of life, functional status, cognitive function, the elderly

## Abstract

**Objectives:**

To investigate the relationship between multimorbidity and health-related quality of life (HRQoL), and explore the effects of functional status and cognitive function on Chinses elderly behind this relationship.

**Methods:**

The Multivariate logistic regression and Tobit regression models were used to determine the influence of multimorbidity on HRQoL. Bootstrap analysis was used to probe the mediating effects of functional status and the moderating role of cognition on multimorbidity and HRQoL.

**Results:**

Results of the 2,887 participants age  ≥ 60  years included in the analysis, 51.69% had chronic diseases. Stroke (*β* = −0.190; 95% confidence interval [CI], −0.232, −0.149; *p* < 0.001) and the combination of hypertension and stroke (*β* = −0.210; 95% CI, −0.259, −0.160; *p* < 0.001) had the greatest influence on HRQoL. Functional status partially mediated the relationship between the number of non-communicable diseases (No. of NCDs) and HRQoL, while cognitive function had a moderating effect not only in the A-path (No. of NCDs to functional status, *β* = 0.143; *t* = 7.18; *p* < 0.001) and but also in the C-path (No. of NCDs to HRQoL, *β* = 0.007; *t* = 6.08; *p* < 0.001).

**Conclusion:**

Functional status partially mediated the relationship between multimorbidity and HRQoL in older adults. And cognitive function, if declined, may strengthen this relationship. These findings suggested that improving cognitive function and functional status in those who developed multimorbidity could be a viable prevention or treatment strategy to improve HRQoL in elderly patients.

## Introduction

Multimorbidity is when multiple chronic diseases occur in the same person. Most studies define it as the presence of two or more chronic conditions (Salisbury et al., 2018). The aging population has increased prevalence of multimorbidity, and a study of US Medicare enrollees estimated that 62% of the population aged 65–74 years developed multimorbidity, and this is expected to increase with age (Lozano-Hernández et al., 2020). A growing number of the studies have reported that elderly people with multimorbidity tend show poor health-related quality of life (HRQoL) and high healthcare costs, although various definition of multimorbidity in different researches (Marengoni et al., 2011; Makovski et al., 2019). Moreover, the effects specific combinations of diseases have on HRQoL and mortality are not always consistent (She et al., 2019). Some combinations are associated with a higher risk of poorer HRQoL and higher mortality because of the mutually affecting coexisting disease progression, such as cardiovascular diseases (She et al., 2019). However, there is still a lack of consensus on the potential mechanisms behind the relationship between multimorbidity and poorer HRQoL and the degree to which this occurs. It is critical to understand the determinants that influence its occurrence to develop better prevention strategies.

At present, patients and health care providers are faced with management of long-term sequelae including cognitive and functional impairment (Rengel et al., 2019). In the disability process model, functional impairment was defined as difficulty performing activities in any domain of life, including activities of daily living (ADL) and instrumental activities of daily living (IADL; Nikolova et al., 2011). Approximately 30–60% of older patients with multimorbidity developed new ADL dependence (Hu et al., 2020). Similarly, patients with multimorbidity are also more likely to show poorer cognitive performance (Kingston et al., 2018). It estimated that the proportion of persons with 4 and over diseases was expected to 67.8% of UK population over 65 years old by 2035, from which two-thirds of those will have cognition impairment and poor quality of life (QoL) (Kingston et al., 2018). A review reported that multimorbidity may interact and then result in functional decline (Calderón-Larrañaga et al., 2019). HRQoL consists of a multidimensional assessment that includes physical, social, and psychological functioning (Jiao et al., 2021). Functional impairment negatively impacts mobility and social participation, and consequently decline HRQoL (Kim et al., 2021). Therefore, functional status may partly mediate the relationship between multimorbidity and HRQoL (She et al., 2019). Notably, compared with older adults keeping normal cognitive function, those who had mild cognitive impairment have decreased physical performance, limited living space activities, and a higher risk of ADL and IADL disability (Dodge et al., 2005). A prospective cohort study found that the combination of somatic multimorbidity and cognitive impairment was 1.34 times (95% confidence interval [CI]: 1.09, 1.64) more associated with ADL/IADL disability than with somatic multimorbidity alone in older adults (Quiñones et al., 2018). In addition, Xiong et al. found that the interaction between multimorbidity and cognitive impairment caused a greater loss of quality-adjusted life expectancy (QALE) than individuals with multimorbidity alone in middle-aged and elderly Chinese population (Xiong et al., 2021). HRQoL utility scores were a necessary index to calculate QALE (Franklin et al., 2022). Taken together, it is plausible that multimorbidity may interact with cognitive impairment to cause functional dependence that consequently contributes to poor HRQoL and may also directly influence HRQoL (Dodge et al., 2005; Marengoni et al., 2011; Kingston et al., 2018; Kim et al., 2021; Xiong et al., 2021; Franklin et al., 2022).

Thus, developing more insight into the underlying mechanisms of how multimorbidity cause poor HRQoL will provide new insights into improving public health guidance for patients with multimorbidity. Our study examined the interrelationships between multimorbidity, functional status, cognitive function, and HRQoL. Based on previous research, the following hypotheses were formulated. Hypothesis 1: Functional status mediates the relationship between multimorbidity and HRQoL. Hypothesis 2: Lower cognitive function is likely to elicit functional decline or poorer HRQoL in patients with multimorbidity. Therefore, it is anticipated that multimorbidity is more strongly associated with functional decline and HRQoL when cognitive function is low. The conceptual frame of the moderated mediation model is manifested in Supplementary Figure 1.

## Sample and methods

### Study design and sample

The data of our study was derived from The Liyang Study (Zhou et al., 2022). In this community-based cohort study, we randomly selected participants from 17 townships in Liyang, Jiangsu, using multi-stage stratified cluster random sampling to select 10,200 residents over 18 years old from March 2019 to June 2020. Firstly, health centers of all towns or streets in Liyang city was selected as the survey sites. Secondly, two communities or villages was randomly selected in each survey site. Thirdly, 300 participants randomly selected in each community or village. Finally, 10,056 participants completed the baseline survey. Our research consisted of 2,887 participants, aged 60 or older after excluding missing of research variables and covariates. All participants provided written informed consent at the recruitment stage in the study. And sampling process and specific inclusion and exclusion criteria was illustrated in Supplementary Figure 2. The more details about study design, research instruments, ethics and data collection were illustrated in our previous article (Zhou et al., 2022). The Life Sciences Ethics Committee of Soochow University approved the protocol for this study (SUDA20211025H02).

### Research instruments

#### HRQoL

The EQ-5D-5L is a universal HRQoL measure from the EuroQoL group which evaluates the current health state of participants. The EQ-5D-5L includes a descriptive system and the EQ visual analog scale (EQ-VAS). The descriptive system consists of five dimensions: mobility (MO), self-care (SC), usual activities (UA), pain/discomfort (PD), and anxiety/depression (AD). Each dimension is described at five levels, corresponding roughly to no, slight, moderate, severe, and extreme problems (Luo et al., 2017). The combination of the various levels of the five dimensions represents different health states. We can estimate the preference-based valuation (EQ-5D index) using the EQ-5D-5L value set for China (Szentes et al., 2020). A higher EQ-5D index indicates better HRQoL (Cronbach’s *α* = 0.809, Kaiser-Meyer-Olkin value (KMO) = 0.791, Bartlett *p* < 0.001).

#### Functional status

Functional status was assessed using the Lawton IADL and the Katz ADL scales. The Lawton IADL scale is composed of six items: housekeeping, cooking food, shopping, telephone use, taking medications, and handling finances (Cronbach’s *α* = 0.915, KMO = 0.874, Bartlett *p* < 0.001; Chen et al., 2015). The Katz ADL scale comprises six basic self-care activities: bathing, dressing, toileting, transferring, continence, and feeding (Cronbach’s *α* = 0.934, KMO = 0.908, Bartlett *p* < 0.001; Connolly et al., 2017). They can all be described at five levels: corresponding roughly to no, mild, moderate, severe, and extreme dependence. Responses to each of the six items in the scale were coded as 1 (from mild to extreme dependence) or 0 (corresponding roughly to no dependence). A higher score indicated more sever functional impairment.

#### Cognitive function

The Abbreviated Mental Test Scores (AMTs) were used to assess cognitive impairment in the elderly. The AMTs is a 10-item screening questionnaire, including age, time of day, year, place, recognition of people, date of birth, national day, president, counting backwards from 20 to 1, and the recall of an address. Each scale was scored with a maximum score of 10. A score between 0 and 3, 4 and 7, and 8 and above suggests severely impaired function, moderately impaired function, and normal cognitive function, respectively (Cronbach’s *α* = 0.854, KMO = 0.856, Bartlett *p* < 0.001; Piotrowicz et al., 2019).

#### Covariates

Participants’ information was gathered including age, gender, educational attainment, marital status, smoking and drinking status. Educational attainment was divided into four categories: illiterate, primary school, middle school, high school or above. Marital status was classified into married or others (widowed, divorced and never married). Smoking status was dichotomized as never smoked (non-smokers), stopped smoking currently (ex-smokers) and currently smoking (current-smokers). Drinking status was classified into currently drinking and no drinking. The number of chronic diseases was the sum of these diseases: hypertension, chronic obstructive pulmonary disease (COPD), stroke, coronary heart disease (CHD), dyslipidemia, asthma, obesity, diabetes, and cancer. And it was grouped into three categories (0, 1, and 2 or above).

### Data analysis

We used mean ± standard deviation (SD), frequency (percentage), cross-tabulations, and graphical displays for descriptive analysis. Statistical analysis of continuous variables was, respectively, performed using the t-test or one-way analysis of variance (ANOVA), and analysis of categorical variables was performed using the chi-square test. We calculated the prevalence of 36 chronic disease pairs using a random pairwise combination of nine diseases. We then selected the top 10 most prevalent chronic disease pairs to explore their effect on HRQoL. The odds ratios (ORs) and 95% confidence intervals (CIs) were calculated using multivariate logistic regression and Tobit regression models, respectively. Spearman correlation coefficients were used to analyze the correlations among the main variables. The Harman single factor test was used to conduct a common method bias test. The PROCESS macro for SPSS was used to perform the mediation and moderated mediation hypotheses (Hayes, 2017). Our study used Model 4 to test the mediated effect, and Model 8 for the moderated mediation analysis. The Bootstrap CIs for the indirect effect were estimated based on 5,000 bootstrap samples and bias-corrected results. Simple slope analysis was used to analyze the moderating effect of cognitive function on the relationship between multimorbidity and HRQoL, as well as functional status. The standardized effects of moderated mediation are presented by the mean center of all continuous variables (Francoeur, 2015). Statistical analyses were performed using SAS version 9.4 software (SAS Institute Inc., Cary, NC, USA), STATA version 16.0 (Stata Corporation, Texas, USA), and SPSS (SPSS Inc., Armonk, NY, USA). The significance level was set at 0.05 (two-sided).

## Results

### Characteristics of the study participants

2,887 participants aged ≥60 years who completed the entire survey. The characteristics of the study participants are shown in Table 1. The mean (SD) age of the 2,887 participants was 71.3 (7.7) years, and 53.0% were female. A total of 51.69% of the participants had chronic diseases, and 17.80% of the study population developed multimorbidity. A limitation in at least one ADL or IADL item was reported in 4.64% and 9.48% of participants, respectively. Cognitive impairment was found in 6.41% of the patients. The mean EQ-5D index score was 0.958 ± 0.104. The most frequently reported problems in the EQ-5D dimension were pain/discomfort (26.29%), followed by anxiety/depression (7.72%), mobility (6.51%), usual activities (6.34%), and self-care (3.64%).

**Table 1:** Baseline characteristics of participants in The Liyang Study.

Characteristics	Total (*n* = 2,887)	Male (*n* = 1,357)	Female (*n* = 1,530)	*p*
Sociodemographic characteristics				
Age mean (SD)	71.3 (7.7)	71.2 (7.7)	71.4 (7.8)	0.460
Education, *N* (%)				
Illiterate	1,028 (35.61)	297 (28.89)	731 (71.11)	<0.001
Primary school	1,034 (35.82)	529 (51.16)	505 (48.84)	
Middle school	650 (22.51)	416 (64.00)	234 (36.00)	
High school or above	175 (6.06)	115 (65.71)	60 (34.29)	
Marital status, *N* (%)				<0.001
Married	2,411 (83.51)	1,220 (50.60)	1,191 (49.40)	
Widowed/divorced/never married	476 (16.49)	137 (28.78)	339 (71.22)	
Smoking, *N* (%)				<0.001
Current	720 (29.94)	703 (97.63)	17 (2.37)	
Former	182 (6.30)	176 (96.70)	6 (3.30)	
Never	1,985 (68.76)	478 (24.08)	1,507 (75.92)	
Drinking, *N* (%)				<0.001
Yes	742 (25.70)	675 (90.97)	67 (9.03)	
No	2,145(74.30)	682 (31.79)	1,463 (68.21)	
Number of chronic diseases, *N* (%)				0.017
0	1,390 (48.31)	662 (47.62)	728 (52.38)	
1	975 (33.89)	480 (49.23)	495 (50.77)	
≥2	512 (17.80)	213 (41.60)	299 (58.40)	
ADL, *N* (%)				0.720
Disabled	134 (4.64)	61 (45.52)	73 (54.48)	
Normal	2,753 (95.6)	1,296 (47.07)	1,457 (52.93)	
IADL, *N* (%)				<0.001
Disabled	284 (9.48)	94 (33.10)	190 (66.90)	
Normal	2,603 (90.16)	1,263 (48.52)	1,340 (51.48)	
Cognitive function, *N* (%)				<0.001
Normal	2,702 (93.59)	1,301 (48.15)	1,401 (51.85)	
Moderately impaired	153 (5.3)	45 (29.41)	108 (70.59)	
Severely impaired	32 (1.11)	11 (34.38)	21 (65.63)	
EQ-5D score mean (SD)	0.958 (0.104)	0.964 (0.099)	0.953 (0.108)	0.002
EQ-5D problems, *N* (%)				
Mobility	188 (6.51)	81 (43.09)	107 (56.91)	0.265
Self-care	105 (3.64)	47 (44.76)	58 (55.34)	0.639
Usual activities	183 (6.34)	72 (39.34)	111 (60.66)	0.032
Pain/discomfort	759 (26.29)	314 (10.88)	445 (58.63)	<0.001
Anxiety/depression	223 (7.72)	90 (40.36)	133 (59.64)	0.040

^a^ ADL, activities of daily living; IADL, instrumental activities of daily living; SD, standard deviation.

Of the chronic diseases in our study, dyslipidemia (57.80%) had the highest prevalence, followed by stroke (54.70%), CHD (48.80%), hypertension (40.42%), and COPD (30.80%; Supplementary Figure 3). Supplementary Table 1 displays the prevalence of 36 chronic disease pairs through a random pairwise combination of nine diseases. The most common multimorbidity combination was diabetes with hypertension (6.51%).

### Multimorbidity and HRQoL

Table 2 illustrates the results of the Tobit regression model of a single chronic disease (Model 1) as well as the top 10 most prevalent multimorbidity combinations (Model 2) on HRQoL. Stroke (*β* = −0.190; 95% confidence interval [CI], −0.232, −0.149; *p* < 0.001; Model 1) was the chronic disease that was found to have the greatest impact on HRQoL. Similarly, stroke with hypertension (*β* = −0.210; 95% CI, −0.259, −0.160; *p* < 0.001; Model 2) was the multimorbidity combination that had the greatest influence on HRQoL.

**Table 2:** Tobit regression results of the effects of single chronic diseases and multimorbidity combinations on EQ-5D index.

Type of disease		*β*	SE	*p*	95% CI
Model 1a	Dyslipidemia	**−0.068**	**0.022**	**0.002**	**−0.054, −0.014**
	Stroke	**−0.190**	**0.021**	**<0.001**	**−0.232, −0.149**
	CHD	**−0.083**	**0.023**	**0.001**	**−0.128, −0.038**
	Hypertension	−0.022	0.011	0.053	−0.045, 0.000
	COPD	**−0.067**	**0.030**	**0.024**	**−0.125, −0.009**
	Cancer	−0.022	0.034	0.526	−0.089, 0.046
	Asthma	**−0.136**	**0.035**	**<0.001**	**−0.204, −0.067**
	Obesity	0.000	0.017	0.980	−0.034, 0.035
	Diabetes	−0.020	0.017	0.243	−0.054, 0.014
Model 2a	DB + HT	−0.020	0.024	0.419	−0.067, 0.028
	OB + HT	−0.011	0.025	0.657	−0.060, 0.037
	OB + CHD	−0.003	0.072	0.961	−0.146, 0.138
	ST + HT	**−0.210**	**0.025**	**<0.001**	**−0.259, −0.160**
	HT + DL	−0.040	0.035	0.246	−0.109, 0.028
	CHD + HT	**−0.086**	**0.032**	**0.007**	**−0.149, 0.024**
	HT + COPD	−0.037	0.040	0.351	−0.115, 0.041
	DB + DL	−0.050	0.050	0.322	−0.147, 0.048
	DB + OB	−0.032	0.050	0.511	−0.131, 0.065
	DL + OB	−0.074	0.053	0.160	−0.178, 0.029

^a^ Tobit regression Model 1 (in single chronic diseases) and Model 2 (of multimorbidity combinations) were established and adjusted for age, gender, education, marital status, smoking, and drinking status. HT, Hypertension; DL, dyslipidemia; COPD, chronic obstructive pulmonary disease; ST, stroke; CHD, coronary heart disease; AS, asthma; OB, obesity; DB, diabetes. The *p* value of all groups applied bold formating were below 0.05.

Compared with fewer chronic diseases and IADL independence, those who developed more chronic diseases and IADL limitations were more likely to experience problems in each EQ-5D dimension and had a lower HRQoL score (Table 3). More disabled ADL items were associated with a lower HRQoL score and increased odds of having problems in some EQ-5D dimensions, such as MO, SC, and UA. Cognitive impairment also had an impact on specific dimensions such as MO, SC, UA, and AD.

**Table 3:** The association of health-related quality of life (HRQoL) and its subdimensions with multimorbidity, functional status, and cognition.

Chronic health conditions	Problems with subdimensions of EQ-5D, OR (95% CI)					EQ-5D index *β* (95% CI) (*n* = 2,887)
	MO (*n* = 188)	SC (*n* = 105)	UA (*n* = 183)	PD (*n* = 759)	AD (*n* = 223)	
No. chronic diseases	**1.49 (1.27,1.74)** ^**^	**1.59 (1.26,2.01)** ^**^	**1.49 (1.27,1.75)** ^**^	**1.31 (1.21,1.43)** ^**^	**1.36 (1.19,1.54)** ^**^	**−0.04 (−0.05,-0.03)** ^**^
No. disabled ADL items	**2.02 (1.62,2.53)** ^**^	**2.33 (1.82,2.99)** ^**^	**1.68 (1.35,2.09)** ^**^	1.08 (0.89,1.31)	0.99 (0.82,1.19)	**−0.05 (−0.07,-0.03)** ^**^
No. disabled IADL items	**1.20 (1.02,1.41)** ^*^	**1.33 (1.10,1.60)** ^**^	**1.49 (1.28,1.74)** ^**^	**1.63 (1.41,1.88)** ^**^	**1.44 (1.24,1.67)** ^**^	**−0.05 (−0.07,-0.04)** ^**^
Cognitive function						
Normal	1.00 (Reference)	1.00 (Reference)	1.00 (Reference)	1.00 (Reference)	1.00 (Reference)	0.00 (Reference)
Abnormal	**2.13 (1.26,3.60)** ^**^	**4.09 (2.10,7.99)** ^**^	**2.44 (1.46,4.07)** ^**^	1.01 (0.70,1.46)	**2.38 (1.53,3.72)** ^**^	0.02 (0.00,0.07)

Each dimension of EQ-5D of the multivariate logistic regression model and EQ-5D index of the Tobit regression model were established and adjusted for age, gender, education, marital status, smoking, and drinking status. *p < 0.05, **p < 0.01. MO, mobility; SC, self-care; UA, usual activities; PD, pain/discomfort; AD, anxiety/depression; CI, confidence interval. The *p* value of all groups applied bold formating were below 0.05.

Correlation analysis showed that the number of chronic diseases was negatively and significantly associated with both functional status (*r* = −0.179, *p* < 0.01) and HRQoL (*r* = −0.013, *p* < 0.01). HRQoL was positively and significantly correlated with both functional status (*r* = 0.034, *p* < 0.01) and cognitive function (*r* = 0.005, *p* < 0.01; Supplementary Table 2). We used the Harman single factor test to conduct a common method bias test. The results suggested six factors had eigenvalues greater than one, and the variance explained by the first factor was 36.70%, which was less than 40% of the critical standard, indicating that there were no serious common method biases in this study.

### Mediation analysis

**Figure 1:**
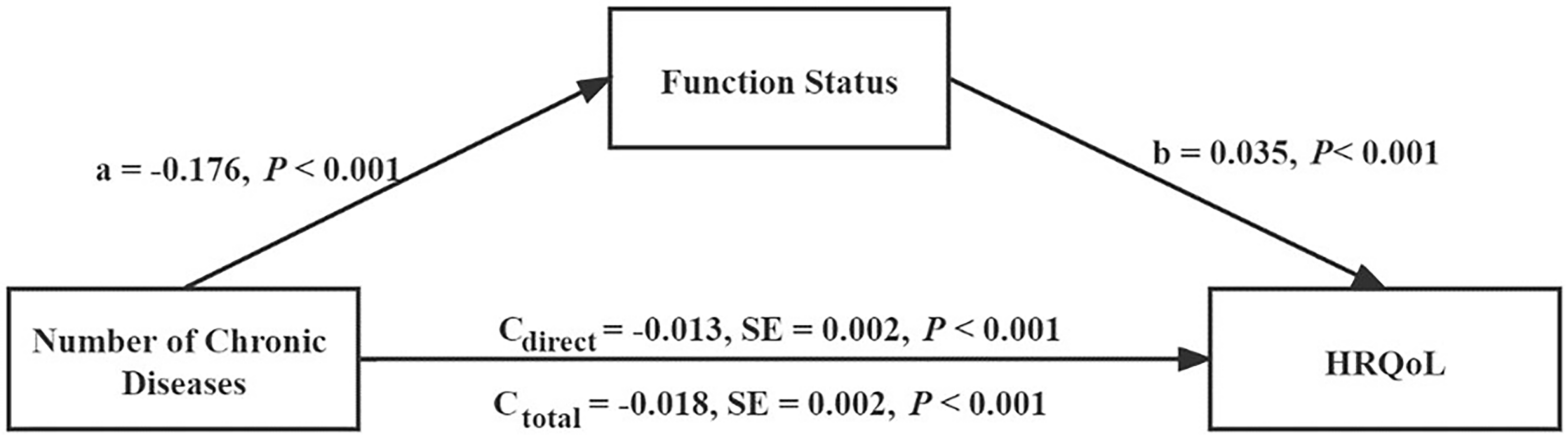
Regression coefficient was derived from a mediation model that was adjusted for age, gender, education, marital status, smoking, and drinking status (*n* = 2,887).

Findings based on the 5,000 bootstrapped samples indicated that when controlling for cognitive condition and other covariates, the number of chronic diseases had a significant negative predictive effect on HRQoL (*β*_total_ = −0.018, SE = 0.002, *p* < 0.001) and that the direct predictive effect of the number of chronic diseases on HRQoL was still significant when the mediating variable functional status was added (*β*_direct_ = −0.013, SE = 0.002, *p* < 0.001; Figure 1). The upper and lower limits of the bootstrap 95% CI for the direct effect of the number of chronic diseases and HRQoL did not include 0, indicating that functional status partially mediated the association between the number of chronic diseases and HRQoL, accounting for 27.78% of the total effect (Table 4).

**Table 4:** Bootstrapped effects of the number of chronic diseases on health-related quality of life (HRQoL) *via* functional status at specific values of cognitive function.

	Cognitive function	Effect	SE	Boot LLCI	Boot ULCI	Mediating rate
Direct effect	−1SD	−0.022	0.002	−0.026	−0.018	
	0^a^	−0.013	0.002	−0.016	−0.010	
	1SD	−0.010	0.002	−0.013	−0.007	
Indirect effect	−1SD	−0.012	0.003	−0.017	−0.006	35.29%
	0^a^	−0.005	0.001	−0.008	−0.003	27.78%
	SD	−0.003	0.001	−0.006	−0.001	23.07%

Standardized effects are presented as mean center of all continuous variables and adjusted for age, gender, education, marital status, smoking and drinking. SE, standardized effect; LLCI, lower limitation confidence interval; ULCI, upper limitation confidence interval.

a Cognitive function have been centered around the mean.

### Moderated mediation analysis

**Figure 2:**
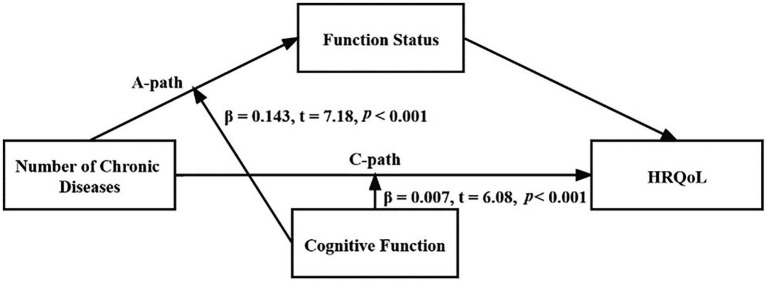
Regression coefficient was derived from a moderated mediation model that was adjusted for age, gender, education, marital status, smoking and drinking status (*n* = 2,887).

**Figure 3:**
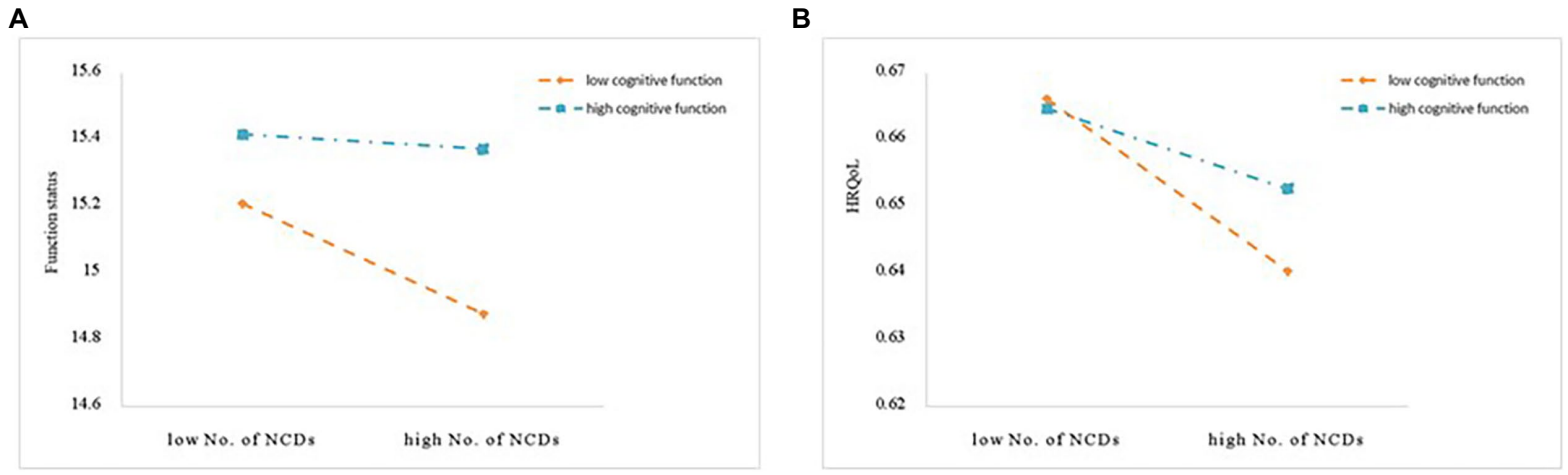
**(A)** Cognitive function moderated the relationship between multimorbidity and functional status. **(B)** Cognitive function moderated the relationship between multimorbidity and HRQoL. No. of NCDs, number of non-communicable diseases; HRQoL, health-related quality of life.

The results of cognitive function at specific values suggested that the effect of cognitive function was lower, while the direct effect, indirect effect, total effect, and mediating rate all increased (Table 4). The moderating effect of cognitive function was seen in the A-path (the number of chronic diseases to functional status) and the C-path (the number of chronic diseases to HRQoL) as shown in Figure 2. The interaction term between the number of chronic diseases and cognitive function was found to significantly predict functional status (*β* = 0.143, *t* = 7.18, *p* < 0.001) and HRQoL (*β* = 0.007, *t* = 6.08, *p* < 0.001), indicating that the effect that the number of chronic diseases had on functional status and HRQoL was dependent on the level of cognitive function. Figure 3 illustrates this effect. HRQoL and functional status were consistently low in patients with lower cognitive function, regardless of the number of chronic diseases they exhibited. The more chronic diseases the patients developed, the worse the HRQoL experienced by the patients. In addition, according to the magnitude of the slope, the predictive effect of the number of chronic diseases on functional status (Figure 3A) and HRQoL (Figure 3B) was stronger if cognitive function declined.

## Discussion

This current study introduces new insights into the body of research that explores the role of chronic disease pairs on HRQoL and the role of multimorbidity, functional status, and cognitive function in HRQoL. Specifically, hypertension with stroke was found to have the greatest negative impact on HRQoL. We confirmed our expectation that functional status partially mediated the association between the number of chronic diseases and HRQoL. In addition, our results supported the assumption that lower cognitive function may strengthen these associations. These findings help increase our outstanding of the potential mechanism behind worse HRQoL in older adults with multimorbidity and help highlight the interventions targeting these risk factors.

### Multimorbidity and HRQoL

We assessed the relationship between common chronic diseases pairs and HRQoL and found that stroke with hypertension had the strongest association with poor HRQoL. In 2016, the global burden disease for risk profiles in the Middle East and North Africa highlight that hypertension was ranked at the first in risk factors for stroke (Jamee Shahwan et al., 2019). The incidence of hypertension was high in our study population, which increased the risk of developing stroke. Stroke is one of the top three global causes of death and long-term disability (Kang et al., 2019). Poststroke disability or low functional status leaves most survivors unable to work, which can lead to serious financial problems and poorer HRQoL (Hsieh and Chiou, 2014). Cumming et al. (2014) showed that cognitive impairment at 3 months post-stroke, and particularly poorer visuospatial ability and attention, were related to poor HRQoL 12 months post-stroke. Therefore, functional impairment and cognitive decline were important risk factors of HRQoL for patients suffering from stroke. However, these evidence in multimorbidity remains sparse with most researches focusing on single diseases. Our research contributed to identifying high risk chronic diseases pairs of poorer HRQoL.

### Mediation effect of functional status

Our results suggested that the effects of multimorbidity on HRQoL were through functional status, which verified our hypothesis 1. The mediation role of functional status in relation to distal variables and in the relationship between multimorbidity and HRQoL is congruent with recent research (Wang et al., 2017; Klompstra et al., 2019; She et al., 2019). A prospective cohort study of community-dwelling older adults (aged 65 years or older) in the United States reported that multimorbidity was linked to functional disability, and lower functional status was found to be a factor related to a lower HRQoL in older people with multimorbidity (Quiñones et al., 2016; Klompstra et al., 2019). Some disease (i.e., stroke and arthritis) are considered as critical factors for functional impairment that is more likely to cause long-term deterioration in self-care ability and physical inactivity (Maresova et al., 2019). With normal physical activities and physiological function restricted and social interaction reduced, lack of mobility assisting technology, increasing number of visits a general practitioner and high healthcare costs of the patient with multimorbidity were easy to reduce their HRQoL (Andersson et al., 2014; Jiao et al., 2021). In addition, it was reported that colorectal cancer or stroke with functional disorders were associated with the lower self-esteem, lower life satisfaction, negative feeling, anxiety and depression, which is expected to decrease the HRQoL (de Tejada et al., 2017; Medhi et al., 2019; Lin et al., 2020). Thus, maintaining functional independence is essential for improving HRQoL in multimorbidity patients.

### Moderation effect of cognition

Our study found that cognitive function played a moderating role in the direct path of the relationship between multimorbidity and HRQoL, which verified our hypothesis 2. There are some potential mechanisms to explain it. On the one hand, Koyanagi et al. reported that patients with multimorbidity generally had a high risk of mild cognitive impairment (Koyanagi et al., 2018). For example, a longitudinal study reported learning (immediate recall) depended on the frontal lobe processes like attention, working memory and executive functioning, which may be more susceptible to cardiovascular diseases (Wei et al., 2020). A literature reported that multimorbidity could synergistically promote cognitive decline such as cardiovascular diseases and arthritis (Vassilaki et al., 2015). On the other hand, cognitive decline could influence the severity and burden of multimorbidity, contributing to the establishment of a vicious circle, which reduced QoL and survial (Calderón-Larrañaga et al., 2019). The decline of cognitive function, and the fear of further deterioration, also directly influences ability of multimorbidity to live independently and take own decisions, consequently declines HRQoL (Hussenoeder et al., 2020).

It is worth noting that our study further found that cognitive function moderated the first half of the mediating model of functional status, which confirmed our hypothesis 2. Namely, multimorbidity in conjunction with lower cognitive function causes functional decline, which consequently contributes to poor HRQoL in older adults. Some literatures may support the potential path mechanisms. Cognitive impairment, was a strong predictor of functional status, and independently mediated functional status difference in traumatic brain injury patients (Dodge et al., 2005; Venkatesan et al., 2021). Loomer et al. (2019) reported that cognitive impairment is related to poorer self-care and mobility function among skilled nursing facilities residents, which in turn increased the risk of functional dependence (Dodge et al., 2005). Functional dependence and cognitive decline occurred simultaneously would accelerate the progression of multimorbidity, which would increase the risk of hospital admission, extended hospital stays, and polypharmacy (Seliger et al., 2015). This would further aggravate the patient’s declining ability to cope with treatment and healthcare burden, which additionally decline HRQoL and increase the risk of premature mortality (Wei et al., 2020). All in all, cognition decline further aggravated the adverse effect of multimorbidity on functional status, consequently strengthened the mediation effect of functional status between multimorbidity and HRQoL in older adults. Our research contributes to elucidating these complicated relationships. Further interventional or experimental studies are need to explore if improving functional status and cognitive function could work as protective factors against multimorbidity. Recently, interventions such as aerobic exercise training combined with cognitive training have been suggested to be effective preventive measures and have the potential to bring significant quality of life benefits for individuals as well as cost savings for healthcare systems (Yeh et al., 2017).

### Strengths and limitations

Our study is the first to use a moderated mediation analysis to simultaneously probe the effects of cognition and functional status on the relationship between multimorbidity and HRQoL. Previous studies only focused on the effect that multimorbidity had on HRQoL, and few have explored the hazard of common pairs of chronic disease to HRQoL in older adults (She et al., 2019). Understanding the common combinations of multimorbidity could help clinicians, researchers, and policymakers to pay attention to more common and severe illnesses. Finally, our research contributes to elucidating the underlying mechanisms between multimorbidity and HRQoL and provide new insights into effective interventions targeted at older patients with multimorbidity.

A potential limitation is that this cross-sectional study could not deduce the causal relationship between multimorbidity and functional status, cognitive function, and HRQoL. We will further explore interventions or experimental studies to substantiate these results. Another limitation is that the diagnosis of all NCDs depended on the self-reporting. However, a study indicated that patient-administered questionnaires might be a reliable source of information for epidemiological purposes in a well-defined chronic disease (Midthjell et al., 1992).

## Conclusion

Our study offers new insights into the relationship between multimorbidity and HRQoL in older adults. Exploring the association between multimorbidity, functional status, cognitive function and HRQoL has shown that functional status partially mediated the relationship between multimorbidity and HRQoL. And cognitive function, if declined, may strengthen this relationship in older adults. These findings suggest that improving cognitive function and functional status in those who developed multimorbidity could be a viable prevention or treatment strategy to improve HRQoL.

## Data availability statement

The raw data supporting the conclusions of this article will be made available by the authors, without undue reservation.

## Ethics statement

The studies involving human participants were reviewed and approved by The Life Sciences Ethics Committee of Soochow University (SUDA20211025H02). The patients/participants provided their written informed consent to participate in this study.

## Author contributions

WH, LZ, LP, LC, and YS designed the study and wrote the protocol. ZF, QH, JC, XC, SL, QH, and NS managed data collection and TL undertook the statistical analysis and wrote the first draft of manuscript. YS and WH gave many valuable comments on the draft and also polished it. All authors contributed to the article and approved the submitted version.

## Funding

This work was supported by the National Natural Science Foundation of China (project number 81973143) and the Priority Academic Program Development of Jiangsu Higher Education Institutions.

## Conflict of interest

The authors declare that the research was conducted in the absence of any commercial or financial relationships that could be construed as a potential conflict of interest.

## Publisher’s note

All claims expressed in this article are solely those of the authors and do not necessarily represent those of their affiliated organizations, or those of the publisher, the editors and the reviewers. Any product that may be evaluated in this article, or claim that may be made by its manufacturer, is not guaranteed or endorsed by the publisher.
