# Supplementary material for: Moderated-mediation analysis of multimorbidity and health-related quality of life among the Chinese elderly: The role of functional status and cognitive function
Source: Front Psychol. 2022 Nov 8;13:978488. doi: 10.3389/fpsyg.2022.978488 (PMC9679780; doi:10.3389/fpsyg.2022.978488)
Supplement: Supplementary file 1 [file Data_Sheet_1.docx]

**Supplementary data:**

**Supplementary Figure1.** The conceptual frame of the moderated mediation model

**Supplementary Figure 2.** Flowchart of sample selection process

**Supplementary Figure 3.** Prevalence (per 100 people) and the cooccurrence of chronic diseases (N=2887)

**Supplementary Table 1.** The prevalence of 36 chronic disease pairs

**Supplementary Table 2.** Pearson correlation coefficients between the variables


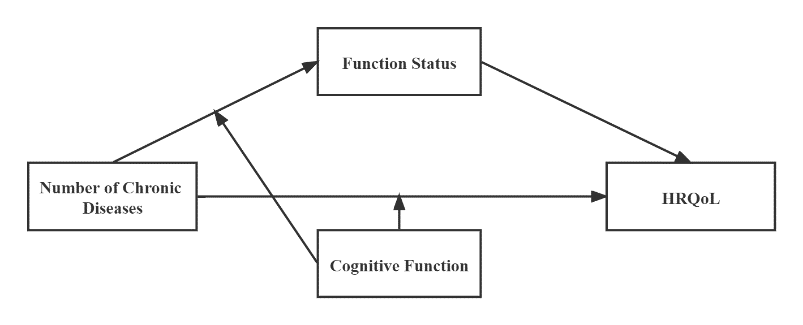


Supplementary Figure1. The conceptual frame of the moderated mediation model


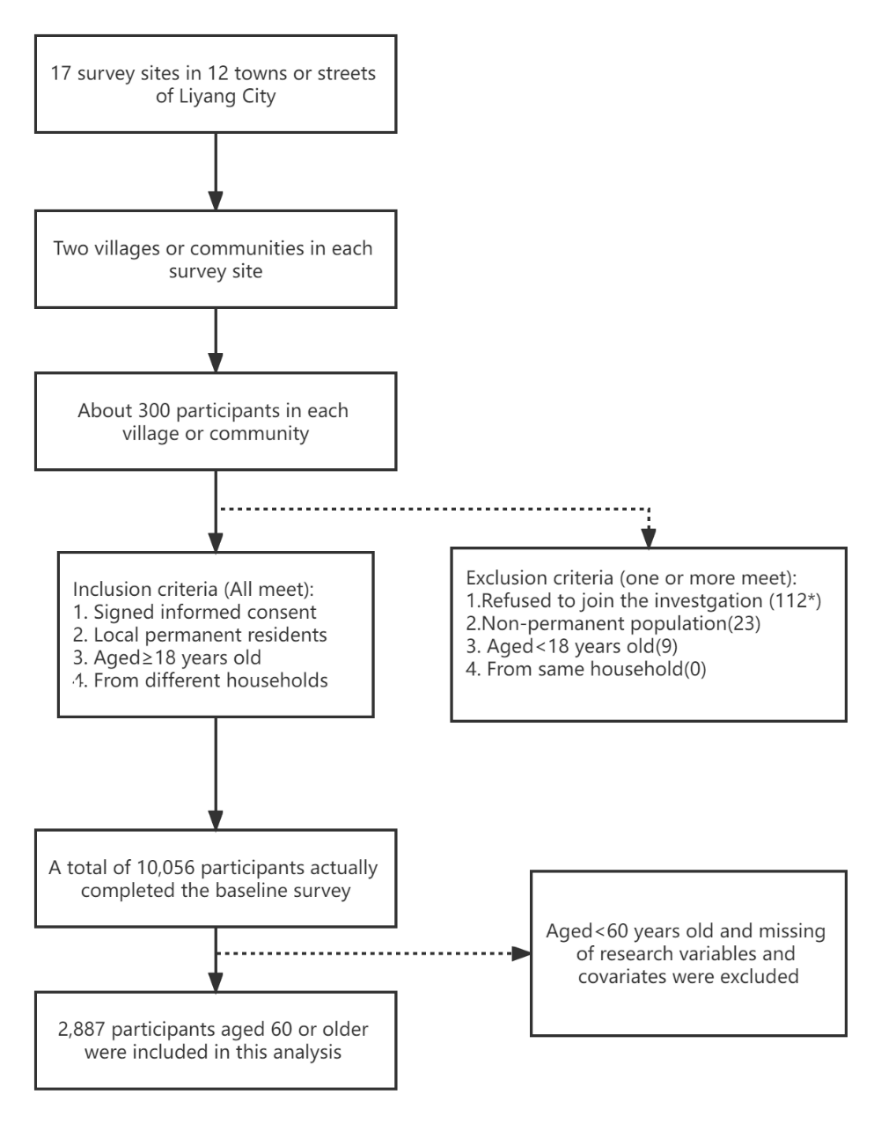


Supplementary Figure 2. Flowchart of sample selection process


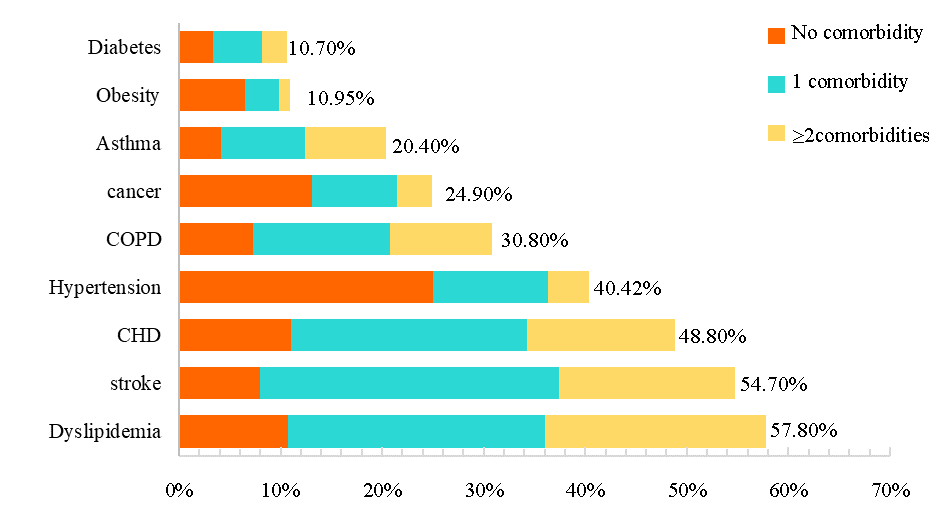


Supplementary Figure 3. Prevalence (per 100 people) and the cooccurrence of chronic diseases (N=2887) (COPD, chronic obstructive pulmonary disease; CHD, coronary heart disease)

Supplementary Table 1. The prevalence of 36 chronic disease pairs

| Type of diseases | prevalence (%) | Type of diseases | prevalence (%) | Type of diseases | prevalence (%) |
| --- | --- | --- | --- | --- | --- |
| DB+HT | 6.51 | DL+ST | 0.94 | DB+COPD | 0.31 |
| OB+HT | 6.34 | DB+CHD | 0.90 | DB+CA | 0.31 |
| OB+CHD | 6.34 | HT+CA | 0.80 | DB+AS | 0.28 |
| ST+HT | 3.84 | ST+OB | 0.80 | ST+COPD | 0.28 |
| HT+DL | 3.81 | CHD+CA | 0.80 | ST+AS | 0.24 |
| CHD+HT | 2.87 | DL+CHD | 0.76 | CHD+AS | 0.24 |
| HT+COPD | 1.73 | COPD+AS | 0.76 | OB+CA | 0.24 |
| DB+DL | 1.56 | ST+CHD | 0.55 | DL+AS | 0.21 |
| DB+OB | 1.49 | CHD+COPD | 0.45 | DL+CA | 0.21 |
| DL+OB | 1.32 | AS+OB | 0.45 | ST+CA | 0.10 |
| HT+AS | 1.25 | DL+COPD | 0.35 | COPD+CA | 0.10 |
| DB+ST | 1.00 | COPD+OB | 0.35 | AS+CA | 0.07 |

*HT, Hypertension; DL, dyslipidemia; COPD, chronic obstructive pulmonary disease; ST, stroke; CHD, coronary heart disease; AS, asthma; OB, obesity; DB, diabetes; CA, cancer

Supplementary Table 2. Pearson correlation coefficients between the variables

|  | Mean | SD | No. of NCDs | Cognition function | Functional status | HRQoL |
| --- | --- | --- | --- | --- | --- | --- |
| No. of NCDs | 0.861 | 0.983 | 1 |  |  |  |
| Cognition function | 9.560 | 1.299 | 0.008 | 1 |  |  |
| Functional status | 11.605 | 1.616 | -0.179^*^ | 0.347^*^ | 1 |  |
| HRQoL | 0.958 | 0.104 | -0.013^*^ | 0.005^*^ | 0.034^*^ | 1 |

No. of NCDs number of non-communicable diseases, HRQoL health-related quality of life, * *P*<0.01.
